# Photothermal Regenerated Fibers with Enhanced Toughness: Silk Fibroin/MoS_2_ Nanoparticles

**DOI:** 10.3390/polym13223937

**Published:** 2021-11-15

**Authors:** Jianjun Guo, Bo Yang, Qiang Ma, Sandra Senyo Fometu, Guohua Wu

**Affiliations:** 1 College of Biotechnology and Sericultural Research Institute, Jiangsu University of Science and Technology, Zhenjiang 212100, China; jianjunguo868@163.com; 2 College of Agriculture, Anshun University, Anshun 561000, China; 3 Anhui Province Key Laboratory of Pollutant Sensitive Materials and Environmental Remediation, Huaibei Normal University, Huaibei 235000, China; byoung92@ustc.edu.cn; 4 Institute of Advanced Technology, University of Science and Technology of China, Hefei 230088, China; 5 College of Environmental and Chemical Engineering, Jiangsu University of Science and Technology, Zhenjiang 212100, China; Qiangma202111@163.com; 6 Biofuels Institute, School of Environment and Safety Engineering, Jiangsu University, Zhenjiang 212013, China; ssfometu@hotmail.com

**Keywords:** regenerated silk fibroin, MoS_2_ nanoparticles, wet spinning, mechanical properties, photothermal

## Abstract

The distinctive mechanical and photothermal properties of Molybdenum sulfide (MoS_2_) have the potential for improving the functionality and utilization of silk products in various sectors. This paper reports on the preparation of regenerated silk fibroin/molybdenum disulfide (RSF/MoS_2_) nanoparticles hybrid fiber with different MoS_2_ nanoparticles contents by wet spinning. The simulated sunlight test indicated that the temperature of 2 wt% RSF/MoS_2_ nanoparticles hybrid fibers could rise from 20.0 °C to 81.0 °C in 1 min and 98.6 °C in 10 min, exhibiting good thermal stability. It was also demonstrated that fabrics made by manual blending portrayed excellent photothermal properties. The addition of MoS_2_ nanoparticles could improve the toughness of hybrid fibers, which may be since the mixing of MoS_2_ nanoparticles hindered the self-assembly of β-sheets in RSF solution in a concentration-dependent manner because RSF/MoS_2_ nanoparticles hybrid fibers showed a lower β-sheet content, crystallinity, and smaller crystallite size. This study describes a new way of producing high toughness and photothermal properties fibers for multifunctional fibers’ applications.

## 1. Introduction

Silk fibroin is a rich natural animal protein that can be reused by dissolving waste silk. A large number of studies have shown that, similar to natural silk, regenerated silk has good biocompatibility, air permeability, and hygroscopicity. Therefore, many researchers are interested in expanding the comprehensive utilization of waste silk products by enriching the function of silk fiber [1,2,3]. Regenerated fibroin fibers with different morphologies and functions can be prepared by wet spinning after silk dissolving. Wet spinning is one of the methods to prepare regenerated silk fibroin (RSF) fibers with different morphologies and functions. However, the mechanical properties of RSF fibers produced by wet spinning are often inferior to natural silk, which seriously affects the application of RSF fibers, so it needs to be improved.

In addition, photothermal materials demonstrate remarkable optical-thermal performance which is an added advantage in textile production, medical instruments, new drug carrier, and manufacturing of military types of equipment [4,5,6,7]. However, so far, there has been little research on the functional regenerated silk with photothermal conversion properties. Improving the quality of textile fabrics has attracted researchers to incorporate photothermal materials into polymer fibers to produce enhanced photothermal fabrics [8,9,10]. Cheng et al. [11] fabricated multifunctional cotton fabrics by coating of anionic waterborne polyurethane (WPU)/Cu_2-X_Se. The WPU/Cu_2-X_Se coated cotton fabrics exhibited high photothermal conversion efficiency, and the coated fabrics also exhibited high photochromic efficiency. The excellent photothermal property and low toxicity of molybdenum disulfide (MoS_2_) nanoparticles (NPs) have rendered it a safer option in textile production [12,13,14,15]. For example, Kumar et al. [16] used MoS_2_ nanosheets to prepare modified poly-cotton masks with excellent antibacterial activity and photothermal properties. Under sunlight, the surface temperature of the modified nano-sheet mask quickly rises to 77 °C, making it self-disinfecting. Zhou et al. [17] engineered Eu^3+^ incorporated MoS_2_ nanoflowers toward efficient photothermal /photodynamic combination therapy of breast cancer. MoS_2_:Eu^3+^ nanosheets also revealed excellent biocompatibility and photostability. Ma et al. [18] evaluated the biological effects of BSA-stabilized gold nanoclusters (BSA-Au NCs) via characterizing the growth status and silk properties of silkworms. BSA-Au NCs showed no significant negative effect on silkworm when the dose of Au was below 9.38 μg/silkworm (about 6.25 mg/kg). In a previous study, functional nanoparticles were directly blended into the spinning solution and RSF fibers were prepared by wet spinning technology which can functionalize RSF fibers and maintain the high strength of RSF fibers as well [19,20,21,22,23,24]. For example, Pham et al. [25] reported a novel approach to toughen epoxy resin with nano-silica fabricated from rice husk using a thermal treatment method with a particle size distribution in a range of 40–80 nm. Our research group has prepared photochromic RSF/WO_3_ NPs fibers with high toughness and photochromic properties under sunlight by wet spinning [26]. MoS_2_ has good light-to-heat conversion performance; however, research has yet to be made in the regenerated silk fibroin complex.

In this work, a series of RSF/MoS_2_ nanoparticles hybrid fibers with different content of MoS_2_ nanoparticles were prepared by wet spinning. The results of a simulated sunlight test indicated that the temperature of 2 wt% RSF/MoS_2_ nanoparticles hybrid fibers could rise from 20.0 °C to 81.0 °C in 1 min and 98.6 °C in 10 min. In addition, the addition of MoS_2_ nanoparticles could improve the toughness of hybrid fibers. We innovatively prepared RSF/MoS_2_ NP_S_ hybrid fibers with high toughness and photothermal properties by wet spinning. Furthermore, we discovered that the addition of MoS_2_ NP_S_ reduced the β-sheet content, crystallinity, and crystallite size in the hybrid fibers, which may be the mechanism of the high toughness of RSF/MoS_2_ NP_S_ hybrid fibers. This study describes a new way of producing high toughness and photothermal properties fibers for multifunctional fibers’ applications.

## 2. Materials and Methods

### 2.1. Materials

Silkworm silk (Chinese Academy of Agricultural Sciences, Zhenjiang, China), Formic acid (FA) (Shanghai Aladdin Co., Ltd., Shanghai, China), sodium carbonate (Na_2_CO_3_) (Sinopharm Group Chemical Reagent Co., Ltd., Beijing, China), anhydrous calcium chloride (CaCl_2_) (Sinopharm Group Chemical Reagent Co., Ltd., Beijing, China), absolute ethanol (C_2_H_5_OH) (Sinopharm Group Chemical Reagent Co., Ltd., Beijing, China), and MoS_2_ nanoparticles (Jiangsu Xianfeng Nanomaterials Technology Co., Ltd., Nanjing, China).

### 2.2. Preparation of Spinning Solution

The silkworm cocoons were added to the Na_2_CO_3_ aqueous solution (0.05 wt%) at a bath ratio of 1:20 and boiled for 30 min; then, the cocoons were rinsed with ultrapure water at 60 °C for 3 times to remove impurities and residual ions. The above experimental process of silk degumming was repeated 3 times. Finally, the degummed silks were dried at 45 °C to a constant weight. The regenerated silk fibroin solution was prepared by dissolving the degummed silk in 5 wt% CaCl_2_-FA solution. Different mass MoS_2_ NPs were added to the silk fibroin solution and stirred for 4 h at 24 °C. The mass ratios of MoS_2_ NPs to degummed silk were 0.2 wt%, 1 wt%, 2 wt%, 3 wt%, 4 wt%, and 6 wt%, respectively.

### 2.3. Wet-Spinning of RSF Solution

A homemade wet spinning device was used in this experiment and all experiments were performed at 24 °C. The spinning solution was poured into a medical syringe and air bubbles were removed by static placement. At 24 °C, the spinning solution in the syringe was squeezed vertically into the coagulation bath by a high-pressure injection pump, where the spinning solution was rapidly condensed into uniform fibers. After stretching treatment, the RSF fibers were placed in 75% ethanol solution for 2 h to remove the residual solvent within the fibers. Finally, the RSF fibers were taken out and dried at 24 °C.

### 2.4. Preparation of Test Samples for Fluorescence Spectroscopy

Furthermore, 80 μL 1 mM Thioflavin T (ThT) solution, 3 mL 10 mg/mL silk fibroin solution, and different mass MoS_2_ NPs were added to a 4 mL tube. The mass ratios of MoS_2_ NPs to silk fibroin were 0.2 wt%, 1 wt%, 2 wt%, 3 wt%, 4 wt%, and 6 wt%, respectively. The samples were cultured at 24 °C for 2 h, 4 h, 6 h, 8 h, 10 h, 12 h, 24 h, respectively, and the fluorescence spectra of the solution were measured [27,28].

### 2.5. Characterization

The morphologies of MoS_2_ NPs and RSF hybrid fibers were observed using a field emission scanning electron microscopy (JSM-IT500HR, Tokyo, Japan) at 20 kV after coating with gold using a sputter coater (SBC-12, Beijing, China). The morphologies of MoS_2_ NPs were observed using transmission electron microscope (Tecnai 12, Philips, Amsterdam, The Netherlands). The structural change of RSF/MoS_2_ NPs hybrid fibers was analyzed by Fourier transform infrared spectroscopy (FTIR, Nicolet iS10, Waltham, MA, USA) with diamond ATR accessories. The structure of MoS_2_ NPs hybrid fibers was analyzed by X-ray diffraction (Rigaku TTR-III, Tokyo, Japan). The radiation source was a copper target (CuKα, λ = 0.1542), and the voltage was 40 kV. The mechanical test of a single fiber of both pristine or RSF hybrid fibers was performed using a mechanical test instrument (Instron 3343, Norwood, MA, USA). The fluorescence test of the fibroin solution was carried out on the F-4600 (Hitachi, Tokyo, Japan). The excitation wavelength is 420 nm, the slit is 5 nm, and the emission wavelength range is 430–700 nm. The photothermal properties of MoS_2_ NPs hybrid fibers were tested by xenon lamp (CEL-HXF300-T3, λ ≥ 420 nm, 300 W, Beijing, China). The thermal degradation of silk fibers was measured by a thermogravimetric analyzer (TGA) (Q5000, TA Instruments, New Castle, DE, USA). The silk fibroin samples were heated from room temperature to 600 °C in N_2_ at a speed of 10 °C/min. The thermogravimetric (TG) curves and the derivative thermogravimetry (DTG) curves were recorded. All tests were performed in triplicate.

## 3. Results and Discussion

### 3.1. Morphology of RSF Fibers and MoS_2_ NPs

**Figure 1:**
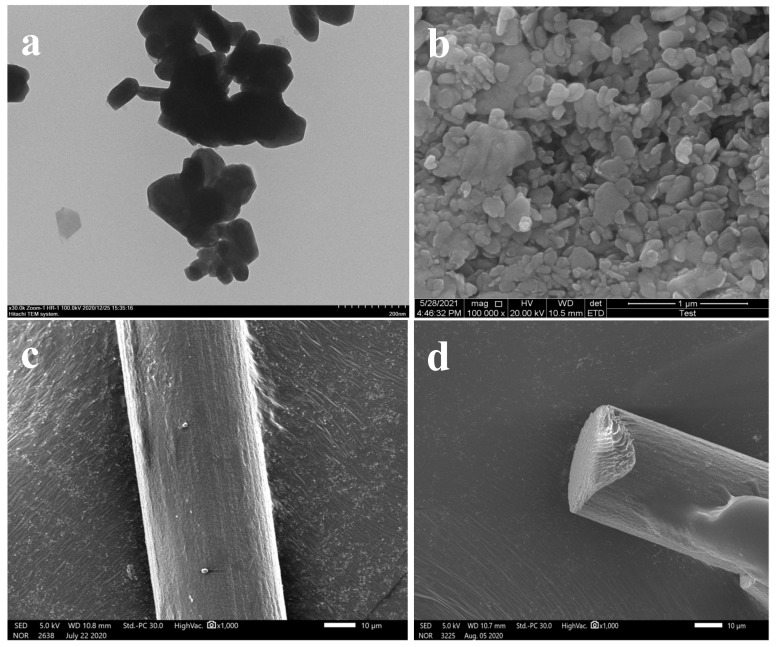
(**a**) TEM image of MoS_2_ nanoparticles; (**b**) SEM image of MoS_2_ nanoparticles. SEM images of 2 wt% RSF/MoS_2_ NPs hybrid fibers; (**c**) surface structure; (**d**) internal structure.

Figure 1a,b showed the TEM and SEM images of MoS_2_ NPs, and it could be seen that MoS_2_ nanoparticles were uniformly dispersed and the particle size was about 90 nm. As shown in Figure 1c,d, the surfaces of 2 wt% RSF/MoS_2_ NPs hybrid fibers were smooth and uniform with no voids and cracks in their internal structure. The average diameter of the fibers was 40.74 ± 1.95 μm. The phenomenon of agglomeration was undetected on the SEM images of RSF hybrid fibers, indicating that the nanoparticles might have good dispersion in the hybrid fibers. In summary, the addition of nanoparticles had no significant impact on the original morphology and characteristics of RSF fibers. These characteristics were the basis of good mechanical properties of the RSF fibers.

**Figure 2:**
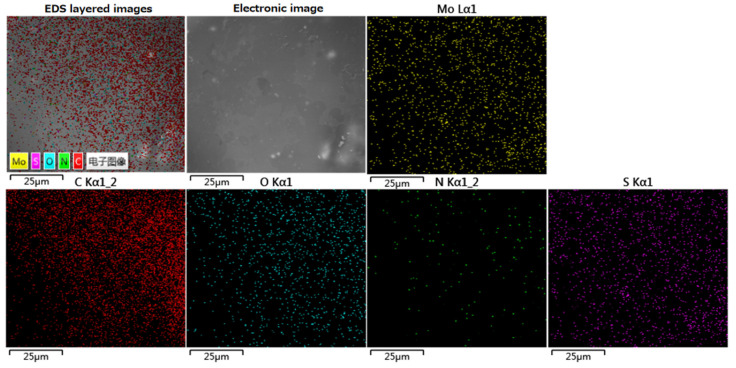
EDS images of 2 wt% RSF/MoS_2_ NP hybrid fiber residues after thermal decomposition.

Figure 2 showed the SEM-EDS element spectrum of the 2 wt% RSF/MoS_2_ NPs hybrid fiber after thermal decomposition. The distribution of C, N, O, S, and Mo elements can be observed, indicating that MoS_2_ NPs were uniformly distributed in the hybrid fiber. Table 1 showed the content of each element in the 2 wt% RSF/MoS_2_ NPs hybrid fibers. C, N, O, S, Mo account for 52.84%, 15.32%, 31.65%, 0.15%, and 0.04%, respectively.

### 3.2. FT-IR Analysis of RSF/MoS_2_ NPs Hybrid Fibers

**Figure 3:**
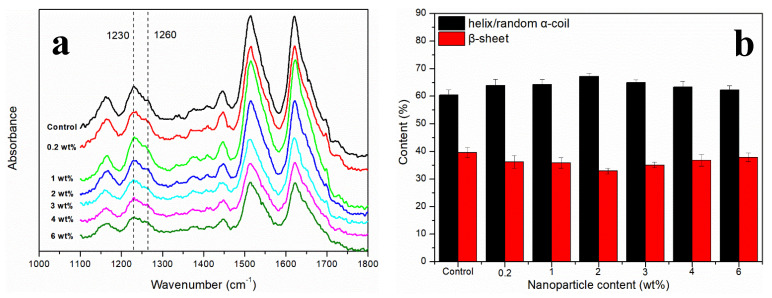
(**a**) FTIR of RSF/MoS_2_ NPs hybrid fibers; (**b**) the contents of the secondary structures of RSF/MoS_2_ NPs hybrid fibers.

It was observed that the infrared spectrum of RSF/MoS_2_ NPs hybrid fibers had no obvious difference when compared with that of RSF fibers (Figure 3a). Both RSF fibers and RSF/MoS_2_ NPs hybrid fibers showed characteristic peaks at 1230 cm^−1^ (α-helix/random coil) and 1260 cm^−1^ (β-sheet) without new absorption peaks, which indicated that the addition of MoS_2_ NPs did not destroy the original secondary structure of RSF.

In this study, deconvolution of the amide III region (1200–1300 cm^−1^) was performed to detect the contents of the secondary structures of RSF [24]. Figure 3b showed the contents of α-helix/random coil (1230 cm^−1^) and β-sheet (1260 cm^−1^) in RSF and RSF/MoS_2_ NPs hybrid fibers. The content of β-sheet of RSF/MoS_2_ NPs hybrid fiber was the lowest when the content of nanoparticles was 2 wt%. This indicates that the incorporation of MoS_2_ NPs hindered the transition from α-helix/random coil to β-sheet, or that was not conducive to the formation of β-sheet. The interaction between silk fibroin and the surface of nanoparticles may lead to structural rearrangement of silk fibroin molecules and affect silk fibroin aggregation during the stirring and spinning process of spinning solutions with MoS_2_ NPs. The binding of nanoparticles to silk fibroin changed the equilibrium constant of β-sheet formation and hindered the formation of the critical nucleus and β-sheet [29]. When the content of MoS_2_ NPs was more than 2 wt%, they might form small-scale agglomeration in RSF, which could not make all the nanoparticles fully in contact with silk fibroin. The size of the MoS_2_ NPs will increase after agglomeration, which means that less self-assembly of silk fibroin was affected by the addition of MoS_2_ NPs.

### 3.3. XRD Analysis of RSF/MoS_2_ NPs Hybrid Fibers

**Figure 4:**
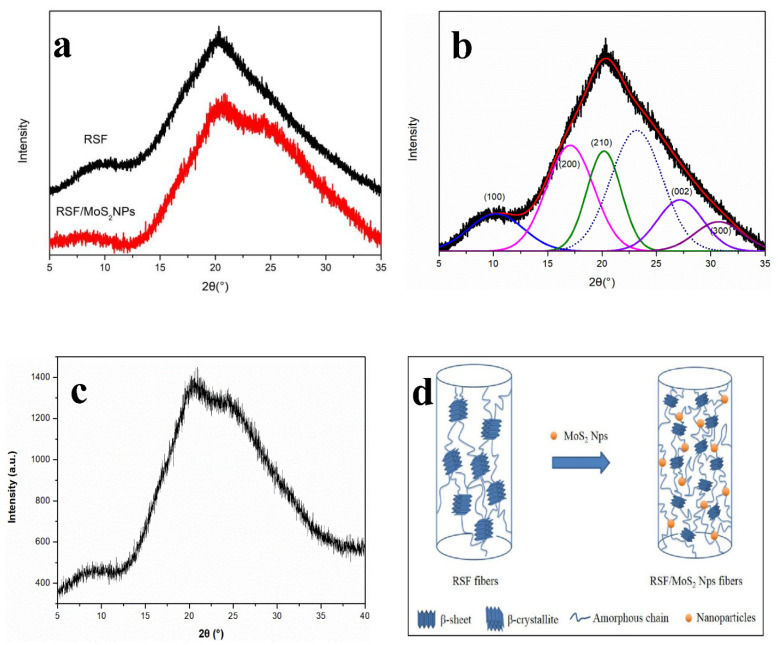
(**a**) XRD pattern of RSF fibers and 2 wt% RSF/MoS_2_ NPs hybrid fibers; (**b**) XRD pattern deconvolution of RSF fibers; (**c**) initial XRD pattern of 2 wt% RSF/MoS_2_ NPs hybrid fibers; (**d**) schematic illustration of structural change of RSF fibers and RSF/MoS_2_ NPs hybrid fibers.

X-ray diffraction (XRD) is a widely used method to study the crystal structure of RSF fibers [30,31,32]. The crystal structures of RSF fibers can be derived by the position and intensity of diffraction peaks. The XRD pattern of RSF fibers and 2 wt% RSF/MoS_2_ NPs hybrid fibers were shown in Figure 4a,c. Furthermore, 2 wt% RSF/MoS_2_ NPs hybrid fibers exhibited the best mechanical properties, so its XRD pattern was selected to compare with that of the RSF fibers. There was no significant difference between the XRD patterns of 2 wt% RSF/MoS_2_ NPs hybrid fibers and RSF fibers. As shown in Figure 4b, the three crystal planes (200), (210), and (002) correspond to the a, b, and c directions. The crystallinity and average β-sheet crystallite sizes were determined by the peak fitting method and calculated by formulas [33,34], respectively. The crystallite size and crystallinity of RSF/MoS_2_ NPs hybrid fibers were shown in Table 2. Compared with RSF fibers, RSF/MoS_2_ NPs hybrid fibers exhibited a lower crystallinity and smaller crystallite size, and this may be due to the chelation and hydrogen bonding interaction between MoS_2_ NPs and RSF, which is not conducive to the formation of β-sheet in RSF (Figure 4d). Therefore, the incorporation of MoS_2_ NPs to silk fibroin might cause the crystallinity of RSF/MoS_2_ NPs hybrid fibers to decrease, which is consistent with the results of FTIR.

### 3.4. Mechanical Analysis of RSF/MoS_2_ NPs Hybrid Fibers

**Figure 5:**
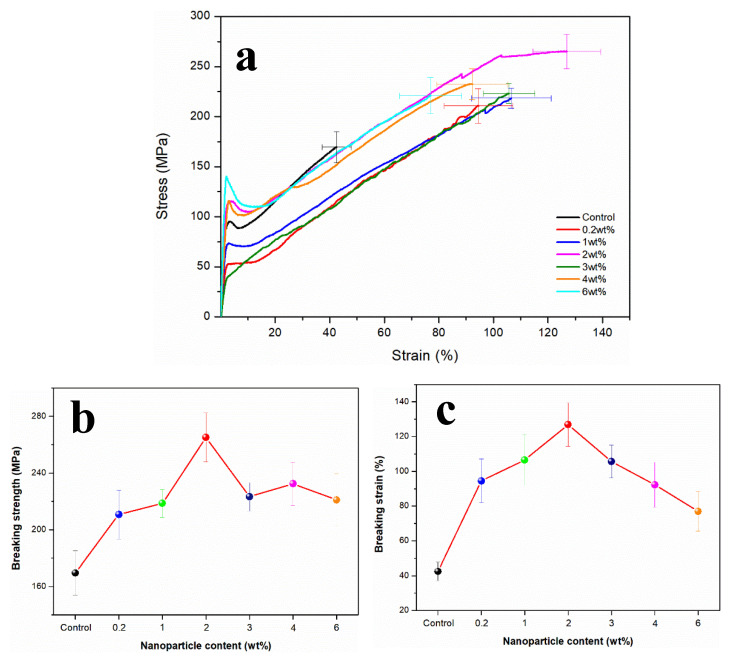
(**a**) Stress–strain curves; (**b**) breaking strength; (**c**) breaking strain of RSF/MoS_2_ NPs hybrid fibers with different MoS_2_ NP contents.

Figure 5 showed the stress–strain curves of RSF fibers and RSF/MoS_2_ NPs hybrid fibers. The 2 wt% RSF/MoS_2_ NPs hybrid fibers exhibited the best mechanical properties when compared to RSF fibers. Its breaking stress (265.04 ± 17.25 MPa) and breaking strain (126.88 ± 12.45%) are higher than those of the blank group (Table 3), respectively, indicating that MoS_2_ NPs could enhance the mechanical properties of the hybrid fibers to a certain extent. This phenomenon suggested that MoS_2_ NPs had a significant toughening effect on the RSF fibers. The addition of MoS_2_ NPs led to a decrease of β-sheet content in RSF fibers, which translated to a decrease in crystallinity and crystallite size. More phase boundaries could be formed by crystal refinement, which has great resistance to plastic deformation and could enhance the strength of the material.

Nova et al. [35] pointed out that the ultimate strength of the spider silk was controlled by the strength of β-sheet nanocrystals, while the strength of β-sheet nanocrystals was directly related to their size. The smaller the crystal inside the fibers, the better its toughness. Keten et al. [36] reported that the crystallite size had a great influence on the mechanical properties of the fibers. The larger crystallite would be destroyed under lower forces, while the smaller crystallite can provide the ability to resist deformation and fracture. The nanoparticles with a small size effect may deflect the crack of RSF fibers during fracture, which can improve the toughness of RSF fibers [37,38]. Meanwhile, we believe that MoS_2_ NPs hindered the formation of larger β-sheet crystals in the fiber, and the decrease of the crystallite size may lead to a more uniform distribution of crystals, which may account for the increase in toughness. The mechanical properties of the fibers began to decline when the content of MoS_2_ NPs was more than 2 wt%, which may be due to the uneven distribution of excessive MoS_2_ NPs content in the fibers, which resulted in the decline of the mechanical properties of the fibers.

### 3.5. Effect of MoS_2_ NPs on the Self-Assembly Behavior of RSF in Solution

**Figure 6:**
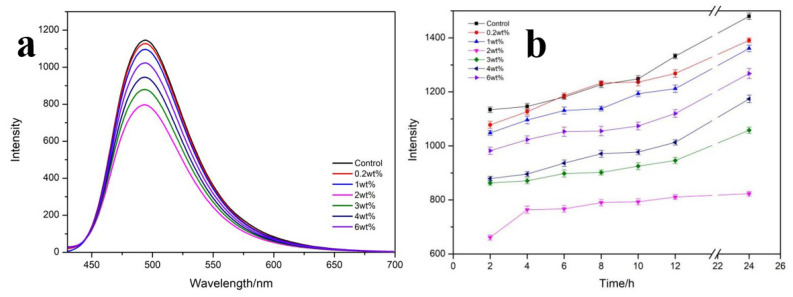
(**a**) The fluorescence emission spectra of RSF/MoS_2_ NPs with different mass fractions at 4 h. (**b**) The fluorescence intensity of RSF/MoS_2_ NPs with different mass fractions changed with time.

Thioflavin T (ThT) is a fluorescent dye that can specifically bind to β-sheet in protein [39]. The fluorescence intensity is proportional to the content of β-sheet in the system, and the probe can be used to detect the content of β-sheet in the system [40]. Figure 6a displayed the fluorescence spectrum of different mass fractions of MoS_2_ NPs mixed with RSF solution for 4 h. The lower the fluorescence value, the lower the β-sheet content, and the weaker self-assembly of β-sheets in the RSF solution. The fluorescence intensity was higher when there was only RSF in the solution. The content of β-sheet in RSF decreased with the addition of MoS_2_ NPs, and the fluorescence intensity was the lowest when the MoS_2_ NPs was 2 wt%, indicating that the content of β-sheet in the solution was the least. This result was consistent with the FT-IR test of RSF/MoS_2_ NPs hybrid fiber.

The fluorescence intensity of RSF/MoS_2_ NPs solution changed with time at different mass fractions in Figure 6b. It was evident that the fluorescence intensity of the 2 wt% RSF/MoS_2_ NPs solution was the lowest at all time points. These results showed that MoS_2_ NPs could hinder and destroy the formation of β-sheet in RSF solution in a concentration-dependent manner.

### 3.6. Thermal Stability of RSF/MoS_2_ NPs Hybrid Fibers

**Figure 7:**
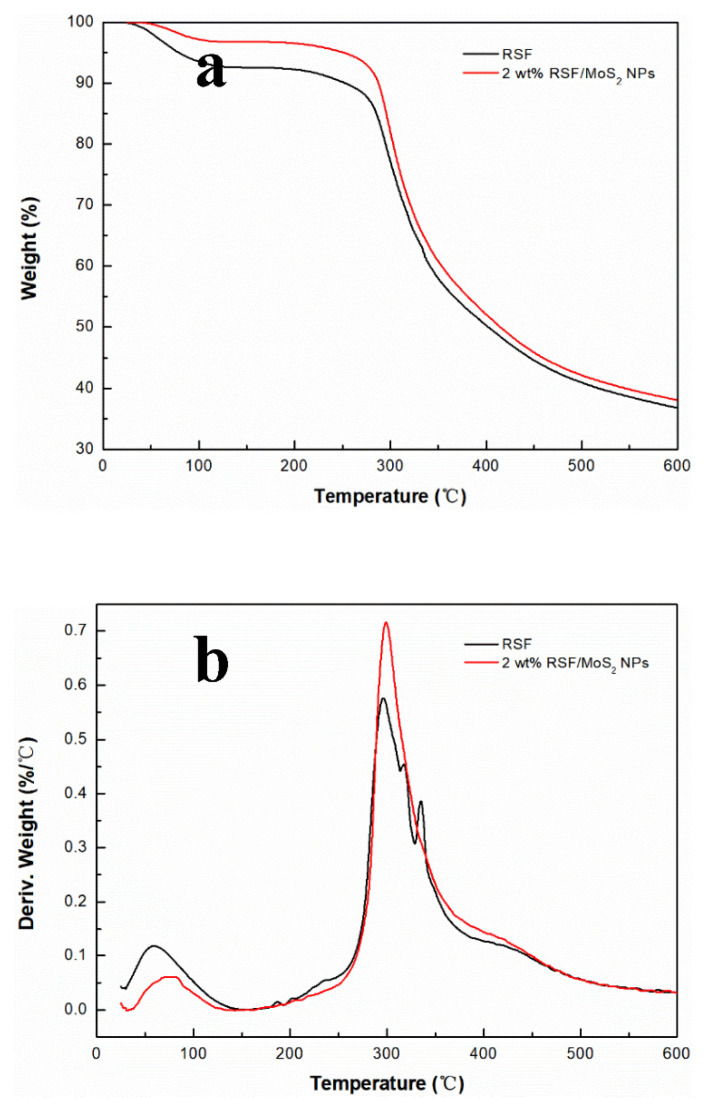
(**a**) TG curves of 2 wt% RSF/MoS_2_ NPs hybrid fibers and RSF fibers; (**b**) DTG curves of 2 wt% RSF/MoS_2_ NPs hybrid fibers and RSF fibers.

For the effect of MoS_2_ NPs on the thermal stability of RSF fibers, TGA was performed on RSF fibers and 2 wt% RSF/MoS_2_ NPs hybrid fibers. Figure 7 displayed the TG curves and their first-order differential curve (DTG) of RSF/MoS_2_ NPs hybrid fibers and RSF fibers. The results showed that RSF and 2 wt% RSF/MoS_2_ NPs hybrid fibers had the same thermal reaction process, and a two-step weight loss was observed. As shown in Figure 7a, the first step was the dehydration stage, and the temperature range was 25–250 °C. The second step was the thermal degradation stage, which occurred at about 280 °C. It can also be seen from Figure 7b that the thermal degradation temperature of RSF fibers was 277.2 °C, while that of 2 wt% RSF/MoS_2_ NPs hybrid fibers increased to 282.6 °C. At 200 °C, compared with the blank group, the weight loss of 2 wt% RSF/MoS_2_ NPs hybrid fibers decreased from 7.9% to 3.6% (Figure 7a). It was demonstrated that the addition of MoS_2_ NPs reduced and delayed the thermal decomposition of the hybrid fibers and improved the thermal stability of the hybrid fibers.

### 3.7. Photothermal Activity of RSF/MoS_2_ NPs Hybrid Fibers and Fabric

**Figure 8:**
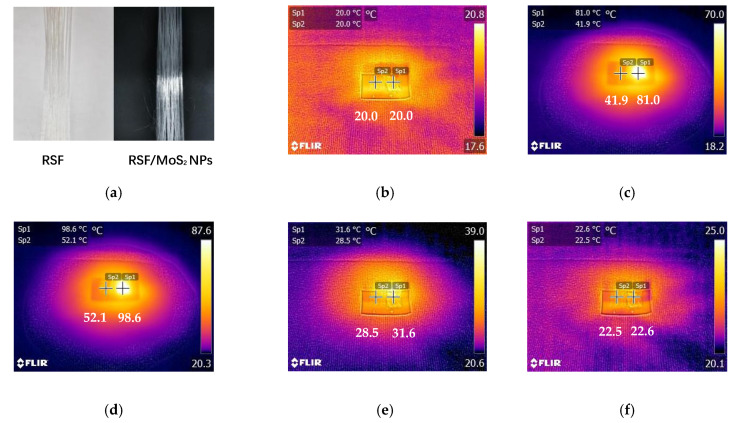
Temperature change of RSF fibers and 2 wt% RSF/MoS_2_ NPs hybrid fibers under the irradiation of simulated sunlight.(**a**) RSF fibers and RSF/MoS_2_ NPs hybrid fibers; (**b**) at the beginning of irradiation; (**c**) after irradiation for 1 min; (**d**) after irradiation for 10 min; (**e**) stop the irradiation for 1 min; (**f**) stop the irradiation for 10 min.

**Figure 9:**
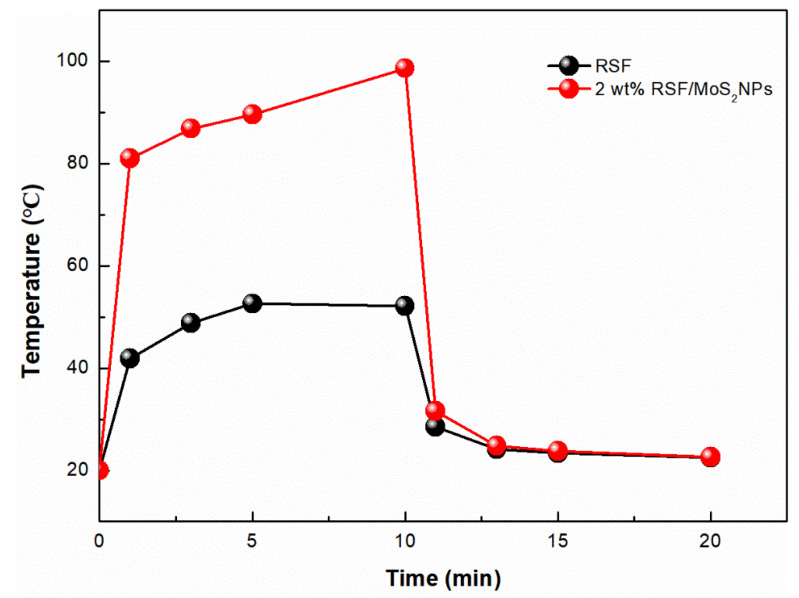
The temperature changes of 2 wt% RSF/MoS_2_ NPs hybrid fibers during heating and cooling.

The objective of this study was to construct a practical photothermal silk fabric. Therefore, it was necessary to test the photothermal properties of RSF/MoS_2_ NPs hybrid fiber and its fabric. Here, the surface temperature of the fibers irradiated by a 300 W xenon lamp (λ ≥ 420 nm) for 10 min and cooled for 10 min after removal of the light source was observed by an infrared imager (Figure 8). Before illumination, there was no difference in temperature between the RSF fibers and 2 wt% RSF/MoS_2_ NPs hybrid fibers (Figure 8b). After simulated illumination, the temperature of 2 wt% RSF/MoS_2_ NPs hybrid fibers increased much faster than that of RSF fibers. The temperature of 2 wt% RSF/MoS_2_ NPs hybrid fibers could rise from 20.0 °C to 81.0 °C in 1 min and 98.6 °C in 10 min (Figure 8b–d), while the temperatures of the RSF fibers were only 41.9 °C and 52.1 °C at these two time points. Figure 9 showed the temperature change of RSF/MoS_2_ NPs hybrid fibers during heating and cooling. It is obvious from Figure 9 that the temperature of hybrid fiber rose faster than that of RSF fiber in the same irradiation time, and its final temperature was also higher. After removing the light source for 10 min, the two fibers returned to almost the same temperature.

**Figure 10:**
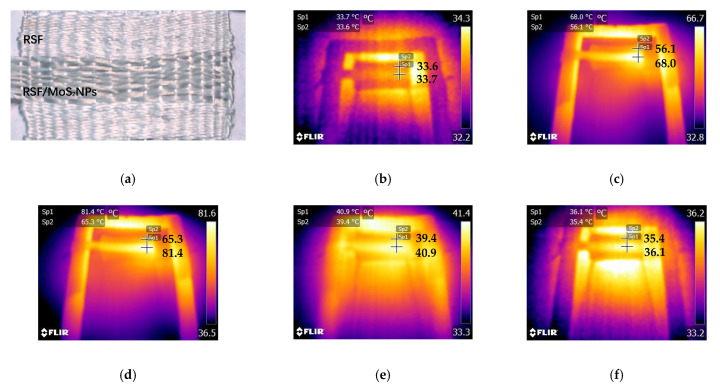
Temperature change of 2 wt% RSF/MoS_2_ NPs woven fabric under the irradiation of simulated sunlight. (**a**) RSF/MoS_2_ NPs fabric; (**b**) at the beginning of irradiation; (**c**) after irradiation for 1 min; (**d**) after irradiation for 10 min; (**e**) stop the irradiation for 1 min; (**f**) stop the irradiation for 10 min.

To test the photothermal effect of 2 wt% RSF/MoS_2_ NPs hybrid fibers in the fabric, hybrid fibers were handwoven into the fabric. Using the same test method, the surface temperature of the fabric was measured by infrared imaging. The temperature of the region made of RSF/MoS_2_ NPs hybrid fibers increased from 33.7 °C to 81.4 °C within 10 min (Figure 10), while the temperature of the region made from RSF fiber only reached 65.3 °C after 10 min irradiation. After cooling for 10 min, their temperatures drop to 36.1 °C and 35.4 °C, respectively (Figure 10f). The results showed that the fabric made of RSF/MoS_2_ NPs hybrid fibers had good photothermal conversion performance in simulated sunlight, and could be used as intelligent photothermal fibers with higher toughness.

## 4. Conclusions

**Figure 11:**
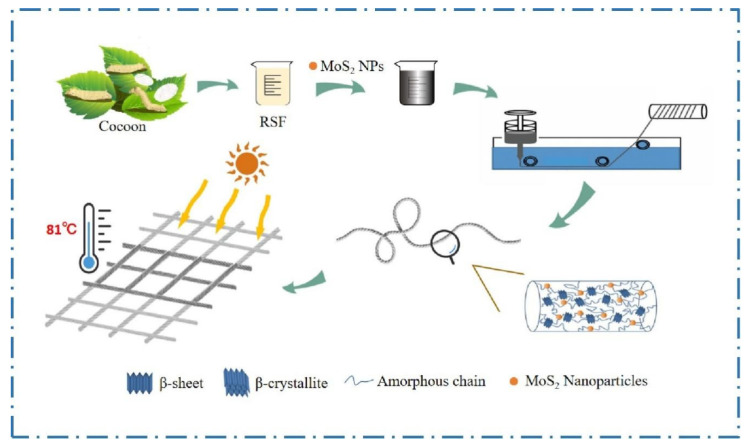
Schematic illustration of RSF/MoS_2_ NPs hybrid fibers with high toughness and photothermal properties.

In this paper, we innovatively prepared RSF/MoS_2_ NP_S_ hybrid fibers with high toughness and photothermal properties by wet spinning (Figure 11). Furthermore, it was discovered that the addition of MoS_2_ NP_S_ reduced the β-sheet content, crystallinity, and crystallite size in the hybrid fibers, which may be the mechanism of high toughness of RSF/MoS_2_ NP_S_ hybrid fibers. The decrease of the β-sheet content was also confirmed in the silk fibroin solution by the Thioflavin T fluorescence spectra test. The mechanical properties of the prepared hybrid fibers were the best when the MoS_2_ NPs concentration was 2 wt%. Meanwhile, both the hybrid fibers and their knitted fabric possessed good photothermal properties. Our research indicated that the RSF/MoS_2_ NPs hybrid fibers could be mass-produced by wet spinning. This study is useful in the production of enhanced textile fabrics required in various product applications.

## Figures and Tables

**Table 1:** Elemental analysis of 2 wt% RSF/MoS_2_ NPs hybrid fibers residues after thermal decomposition.

Elements	The Relative Percentage Contents/%
C	52.84
N	15.32
O	31.65
S	0.15
Mo	0.04

**Table 2:** Structural parameters of RSF/MoS_2_ NPs hybrid fibers.

Sample	Crystallinity/%	L_hkl_ nm			V/nm^3^
		(200)	(210)	(002)	
Control	41.32	1.93	2.44	1.39	6.55
RSF/MoS_2_ NPs-2 wt%	32.66	1.57	2.16	1.25	4.24

**Table 3:** The mechanical properties of RSF/MoS_2_ NPs hybrid fibers.

Sample	Breaking Strength/MPa	Breaking Strain/%
Control	169.58 ± 15.57	42.50 ± 5.34
RSF/MoS_2_ NPs-0.2 wt%	210.77 ± 17.26	94.48 ± 12.57
RSF/MoS_2_ NPs-1 wt%	218.53 ± 9.85	106.57 ± 14.56
RSF/MoS_2_ NPs-2 wt%	265.04 ± 17.25	126.88 ± 12.45
RSF/MoS_2_ NPs-3 wt%	223.22 ± 10.03	105.70 ± 9.45
RSF/MoS_2_ NPs-4 wt%	232.39 ± 15.24	92.26 ± 12.98
RSF/MoS_2_ NPs-6 wt%	220.98 ± 18.25	76.94 ± 11.34

## Data Availability

The data presented in this study are available on request from the corresponding author.
